# Tre2-Bub2-Cdc16 Family Proteins Based Nomogram Serve as a Promising Prognosis Predicting Model for Melanoma

**DOI:** 10.3389/fonc.2020.579625

**Published:** 2020-10-28

**Authors:** Ling Tang, Cong Peng, Su-Si Zhu, Zhe Zhou, Han Liu, Quan Cheng, Xiang Chen, Xiao-Ping Chen

**Affiliations:** ^1^Department of Clinical Pharmacology, Xiangya Hospital, Central South University, Changsha, China; ^2^Institute of Clinical Pharmacology, Central South University, Hunan Key Laboratory of Pharmacogenetics, Changsha, China; ^3^Hunan Key Laboratory of Skin Cancer and Psoriasis, Xiangya Hospital, Central South University, Changsha, China; ^4^Department of Dermatology, Xiangya Hospital, Central South University, Changsha, China; ^5^Hunan Engineering Research Center of Skin Health and Disease, Xiangya Hospital, Central South University, Changsha, China; ^6^Department of Neurosurgery, Xiangya Hospital, Central South University, Changsha, China

**Keywords:** melanoma, Tre2-Bub2-Cdc16, nomogram, prognostic, model

## Abstract

Tre2-Bub2-Cdc16 (TBC) proteins are conserved in eukaryotic organisms and function as negative feedback dominating the GAPs for Rab GTPases, while the function of TBC proteins in melanoma remains unclear. In this study, we observed the differential expression of 33 TBC genes in TCGA datasets classified by clinical features. Seven prognostic-associated TBC genes were identified by LASSO Cox regression analysis. Mutation analysis revealed distinctive frequency alteration in the seven prognostic-associated TBCs between cases with high and low scores. High-risk score and cluster 1 based on LASSO Cox regression and consensus clustering analysis were relevant to clinical features and unfavorable prognosis. GSVA analysis showed that prognostic-associated TBCs were related to metabolism and protein transport signaling pathway. Correlation analysis indicated the relationship between the prognostic-associated TBCs with RAB family members, invasion-related genes and immune cells. The prognostic nomogram model was well established to predict survival in melanoma. What’s more, interference of one of the seven TBC proteins TBC1D7 was confirmed to inhibit the proliferation, migration and invasion of melanoma cells *in vitro*. In summary, we preliminarily investigated the impact of TBCs on melanoma through multiple bioinformatics analysis and experimental validation, which is helpful for clarifying the mechanism of melanoma and the development of anti-tumor drugs.

## Introduction

Melanoma is the most aggressive skin tumor that accounts for 90% of the deaths caused by skin cancer (1). The incidence has been continuously rising over the past decades with about 232,100 new cases and 55,500 deaths annually. In 2012, the world standard incidence fluctuated from 0.2 in southeast Asia to 7.7 in America, 10.2 in EU and 13.8 in North America every 100,000 people years (2). Multiple risk factors have been known to be associated with risk for melanoma such as hair, heteromorphic nevus, family history, age, gender, and increased exposure to ultraviolet radiation (3, 4). At the early stage of tumor progression, surgical resection is the primary approach to convincing a good prognosis. Once it progresses to stages of metastasis, it’s hard to cure owing to therapy resistance and recurrence in spite of the application of targeted therapy and immunotherapy (5, 6). There is an urgent need to clarify the complexity of pathogenesis during the progression and treatment of melanoma.

Proteins containing the Tre2-Bub2-Cdc16 (TBC) domain belong to the Rab-specific GTPase-activating proteins (GAPs) that are highly conserved in eukaryotic organisms (7). These proteins regulate the GAPs for Rab GTPases that control the production of cytokinesis through negative feedback mechanisms. The family includes 44 predicted proteins and regulates multiple cellular biological processes such as obesity, epilepsy, allergic dermatitis and cancer (8). Knowledge about the function of the TBC family is growing. For examples, TBC1D4 is an Akt substrate and participates in the transport of glucose transporter 4 (GLUT4) to the plasma membrane (9, 10). TBC1D3 (also referred to as PRC17) is overexpressed in prostate cancer and its overexpression promotes the growth of NIH3T3 cells (11). TBC1D7, the GAP for Rab17, can significantly promote the growth of lung cancer cells, and an obvious correlation between the expression of TBC1D7 and poor prognosis is observed (12). TBC1D16 is identified as a driver of melanoma and promotes the growth of melanoma cells (13). However, the function of TBC family proteins in melanoma is largely unknown.

In this study, we conducted a comprehensive analysis of 33 TBC family members and their possible roles in predicting disease prognosis in melanoma through bioinformatics tools and experimental validation. These data showed that seven prognostic-associated TBCs engaged in the progression of melanoma. A prognostic nomogram model based on the seven prognostic-associated TBCs was well established in predicting survival, providing novel insights into the diagnosis and therapies in melanoma.

## Materials and Methods

### Datasets Analysis

The training set from the Cancer Genome Atlas (TCGA) database with clinical information and the validation set of the GEO database (GSE65904) for melanoma patients were downloaded, the clinical information of the patients was shown in the **Supplementary Table 1**.

### Least Absolute Shrinkage and Selection Operator (LASSO) Cox Regression Analysis

The risk assessment model based on TBCs was constructed by LASSO Cox regression analysis. A total of 33 TBC members were introduced to count the coefficients of LASSO based on the highest value of lambda described previously (14). The formula of risk score was created based on its coefficients in multivariate cox regression analysis and the expression values of the seven prognostic-associated TBC genes. Melanoma patients in TCGA dataset were grouped into low-risk group and high-risk group based on the cut-off point of median risk score.

### Alterations of Genes in Melanoma

To explore the genetic alterations in melanoma, melanoma samples were grouped into low-risk and high-risk groups based on the risk scores. The mutation frequency of the top 20 genes in the low- and high-risk groups were counted and displayed, the data of copy number variations and Human genome reference consortium h19 were downloaded from the TCGA and GISTIC 2.0, respectively (15). Mutation frequencies of the seven prognostic-associated TBCs identified in our study was also analyzed.

### Consensus Clustering Analysis

Melanoma samples were divided into unique groups through consensus clustering analysis with the R language “Consensus Cluster Plus” as reported elsewhere (16). The number of clusters named K ranging from 2 to 10, and the supreme number of clusters was decided based on the cumulative distribution curves and consensus matrices (17). The difference in the expression of TBC genes and clinical traits between two clusters were displayed with heat map.

### Survival Analysis

Melanoma samples from the TCGA dataset were divided into groups (high-risk and low-risk or cluster 1 and cluster 2) based on risk scores or clusters. The Kaplan-Meier curve was used to compare the overall survival (OS), progression-free interval (PFI), and disease-specific survival (DSS) between the groups. Additionally, distant-metastasis survival (DMS) and DSS in GEO (GSE65904) patients set were also analyzed.

### Gene Set Variation Analysis (GSVA)

The R language “GSVA” was used to execute the functional enrichment analysis to clarify the unique signaling pathways of TBC families in melanoma, the cutoff value of |correlation coefficient| > 0.5 was defined (18). Gene Ontology (GO) and Kyoto Encyclopedia of Genes and Genomes (KEGG) pathway analysis were carried out with the help of the Molecular Signatures Database (MSigDB) (19). The correlation between TBCs and RABs family members that are involved in protein transport and key genes relevant to the hallmarks of cancer including invasion and epithelial-mesenchymal transition (EMT) was also investigated.

### Prognostic Nomogram Construction

The prognostic model was established according to univariate and multivariate Cox regression analyses with *P* < 0.05. A nomogram was constructed using **“**RMS package” (20) to predict 5- and 10-year OS based on the results produced based on Cox regression analyses, and the prediction precision of the prognostic nomogram for OS was assessed by the calibration of the area under the curve (AUC).

### Delineation of the Receiver Operating Curve (ROC)

ROC and AUC were used to observe the predicted performance of TBCs, risk scores and clustering in multiple clinical traits including 5- year OS, 10- year OS and subtypes.

### Cell Lines and siRNA Transfection

Melanoma cells A375 and Sk-Mel-28 purchased from American Type Culture Collection (ATCC) which were cultured in Dulbecco’s modified Eagle’s medium (BI, Israel) with 10% fetal bovine serum (FBS) (BI, Israel) at 37°C in 5% CO2. 100nM siRNA purchased from RIBO Biotechnology was transfected into for 24–72 h using turbofect (Thermofisher, USA) according the manufacturer’s instruction.

### Quantitative Real-Time PCR

RNA was extracted from melanoma cells A375 and Sk-Mel-28 interfered with si-TBC1D7 and NC as control. cDNA was synthesized for real-time PCR adopting SYBR Green qPCR mix (CWBiotech, China). The primers are listed as following: TBC1D7-Forward: GAGTCCCATGCCAAGGTGATGATG; TBC1D7- Reverse: TGCGGAGATAGACTTCAGCCTGAG; GAPDH- Forward: CTTTGGTATCGTGGAAGGACTC; GAPDH- Reverse: AGTAGAGGCAGGGATGATGT.

### Cell Proliferation

Melanoma cells 2.5×103 A375 and Sk-mel-28 interfered with si-TBC1D7 were seeded into 96 wells and cultured with complete medium for 24, 48, and 72 h. Cell proliferation was detected with CCK-8 kit according the manufacturer’s instruction at 450 nM wavelength, the OD values were detected with microplate reader (BioTek).

### Migration and Invasion

For migration, 1×10^4^ melanoma cells were seeded into the upper chambers (Corning, USA) with 550ul medium containing 30% FBS put into the lower chambers and cultured for 24 h. Similarly, chambers with Matrigel (Corning, USA) embedded were used to detect invasion with the number of 5×10^4^ cells. After culturing for 30 h, cells in the upper chambers were removed, transwell chambers fixed with 4% paraformaldehyde were stained with 0.5% crystal violet. five random fields of vision were captured and counted.

### Statistical Analysis

All the analytic methods were carried out with the R package (version 3.5.3). Two-tailed Students’ *t*-test was used to analyze the difference between two subgroups, while the comparison of multiple subgroups was assessed by a one-way ANOVA. The prognostic value based on risk scores and clinical traits was analyzed by univariate and multivariate Cox regression analysis. The algorithm of partition around medoids (PAM) was introduced into consensus clustering analysis. The clinical features’ discrepancy between clusters was assessed by the Chi-squared test. Comparison of OS, PFI, DSS and DMS between subgroups (low-risk vs high-risk score, cluster 1 vs cluster 2) was carried out by Kaplan-Meier, and correlation analysis was determined by Spearman’s rank correlation coefficient. *P*< 0.05 was regarded as statistical significance. The Schoenfeld test was used to evaluate the proportional hazards (PH) assumption.

## Results

### Relationship Between the mRNA Expression of TBCs and Clinical Traits in Melanoma

**Figure 1:**
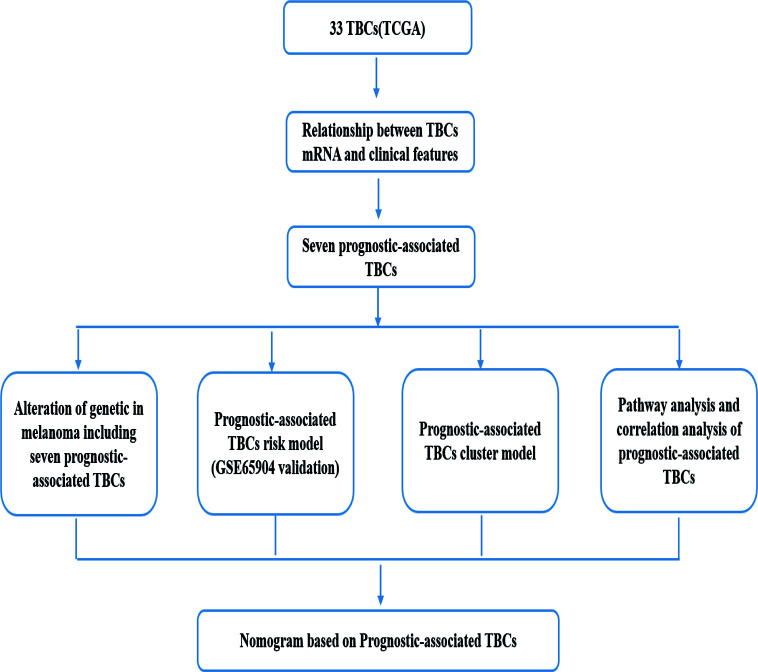
A flowchart designed for the study.

**Figure 2:**
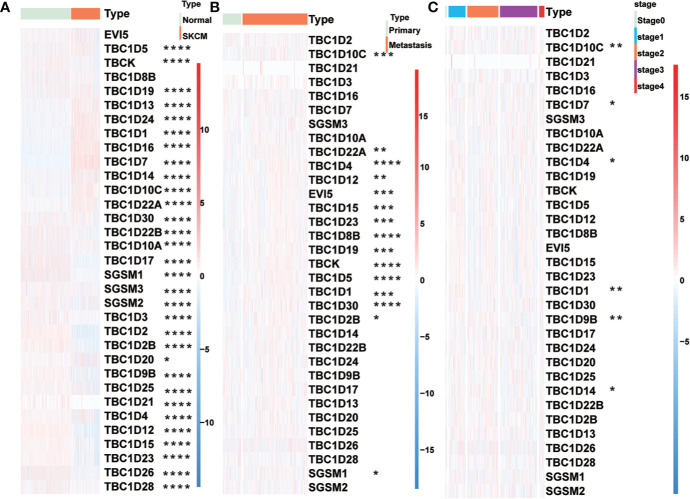
Relationship between TBCs mRNA expression and clinical features in melanoma. The heat maps based on clustering analysis according to the subgroups exhibited the differential expression patterns of TBCs (Tumor type: Normal vs Tumor **(A)**, Tumor type: Primary vs Metastasis **(B)** and Tumor stage: stage0 vs stage1 vs stage2 vs stage3 vs stage4 **(C)**. *p < 0.05, **p < 0.01, ***p < 0.001, **** < 0.0001.

The workflow designed for the study was displayed in **Figure 1**. A total of 33 TBC family members were shown in the TCGA dataset. mRNA expression levels of some of the TBCs genes were closely related to some clinical traits of melanoma such as tumor type, tumor status, age, gender and stage. Specially, we observed significantly increased mRNA expression level for 10 *TBC* genes (*TBC1D5*, *TBC1D19*, *TBC1D13*, *TBC1D24*, *TBC1D1*, *TBC1D16*, *TBC1D7*, *TBC1D14*, *TBC1D10C*, and *TBC1D22A*), and decreased mRNA expression level for 21 *TBC* genes (*TBCK*, *TBC1D30*, *TBC1D22B*, *TBC1D10A*, *TBC1D17*, *SGSM1*, *SGSM2*, *SGSM3*, *TBC1D3*, *TBC1D2*, *TBC1D2B*, *TBC1D20*, *TBC1D9B*, *TBC1D25*, *TBC1D21*, *TBC1D4*, *TBC1D12*, *TBC1D15*, *TBC1D23*, *TBC1D26*, and *TBC1D28*) in skin cutaneous melanoma (SKCM) compared with normal tissues (**Figure 2A**). The mRNA expression levels of 15 genes (*TBC1D10C, TBC1D22A, TBC1D4, TBC1D12, EV15, TBC1D15, TBC1D23, TBC1D8B, TBC1D19, TBCK, TBC1D5, TBC1D1, TBC1D30, TBC1D2B* and *SGSM1*) was increased in cases with metastasis as compared with those only with the primary focuses (**Figure 2B**). According to the clinical stage, differences in the mRNA expression of *TBC1D10C, TBC1D7, TBC1D4, TBC1D1, TBC1D9B*, and *TBC1D14* were also observed (**Figure 2C**). Furthermore, difference between patients stratified by neoplasm cancer status (with tumor vs tumor free) registered was also analyzed. We observed a higher mRNA expression of *TBC1D10C, TBC1D4, TBC1D12, TBCK, TBC1D5, TBC1D30* and a lower expression of *TBC1D24* in patients with tumor compared with tumor free patients (**Supplementary Figure 1A**). We also observed differential mRNA expression of *TBC1D10C, TBC1D19, TBC1D5, TBC1D12, EV15, TBC1D23, TBC1D17*, and *TBC1D7* in tumor tissue sites (**Supplementary Figure 1B**). Age seemed to have no effect on the mRNA expression of the majority of the TBC family members in melanoma except for *TBC1D4, TBC1D1, TBC1D25*, and *TBC1D10A* (**Supplementary Figure 1C**).

### Identification of Seven Prognostic-Associated TBC Genes in Melanoma Based on LASSO Cox Regression Analysis and Somatic Mutations of These Genes in Melanoma

**Figure 3:**
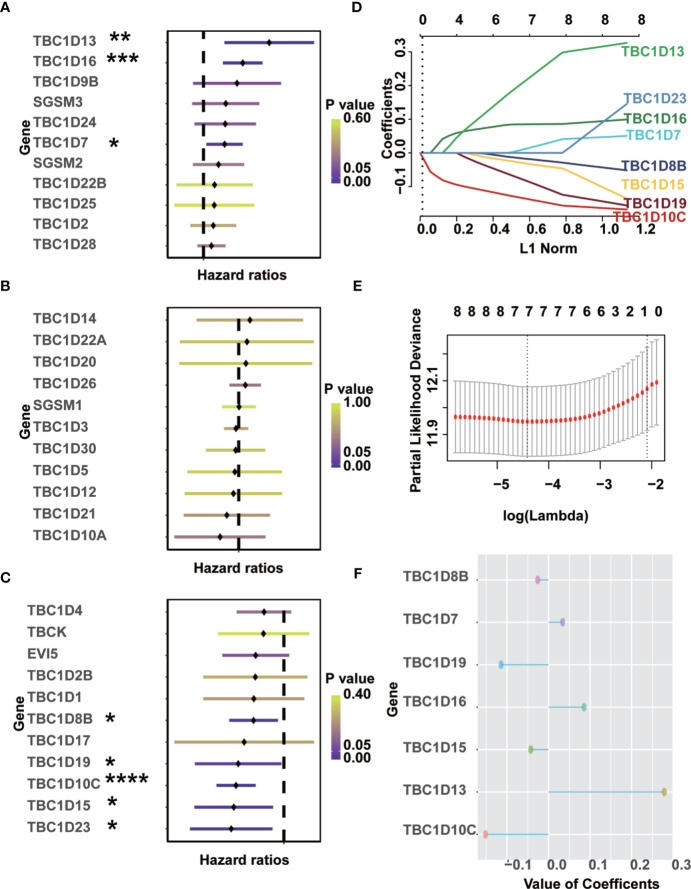
Identification of prognostic-associated TBCs from TCGA. **(A–C)** Univariate Cox regression analyses were finished to screen prognostic-associated TBCs from TCGA. **(D–F)** LASSO coefficients of the seven prognostic-associated TBCs were calculated and displayed. *p < 0.05, **p < 0.01, ***p < 0.001, **** < 0.0001.

Univariate Cox regression analysis was performed to identify prognostic-associated TBCs genes with melanoma samples from the TCGA dataset. As shown in **Figures 3A–C**, 8 of the 33 *TBC* genes showed marked prognostic value (*p *< 0.05). The prognostic-associated genes were further analyzed with the LASSO Cox regression model and the minimum partial likelihood deviance after cross-validation was adopted to identify the optimal lambda value. Seven prognosis-associated *TBC* genes including *TBC1D13*, *TBC1D16*, *TBC1D7*, *TBC1D8B*, *TBC1D15*, *TBC1D19*, and *TBC1D10C* were identified (**Figures 3D–F**). Comparison of the transcription levels of the seven prognostic-associated TBC genes with the TCGA melanoma dataset showed that the relative mRNA expression of 5 gens (*TBC1D10C*, *TBC1D19*, *TBC1D16*, *TBC1D13* and *TBC1D7*) were significantly upregulated, 1 (*TBC1D15)* was significantly downregulated, while 1 (*TBC1D8B*) was comparable in the SKCM as compared with the normal tissues (**Supplementary Figure 2A**). Changes in protein expression levels of these 7 representative TBCs in melanoma with immunohistochemistry staining data based on the online Human Protein Atlas was also analyzed. As shown in **Supplementary Figures 3A–D**, protein expression of TBC1D10C, TBC1D19, TBC1D16, and TBC1D13 were increased significantly in melanoma tissues.

**Figure 4:**
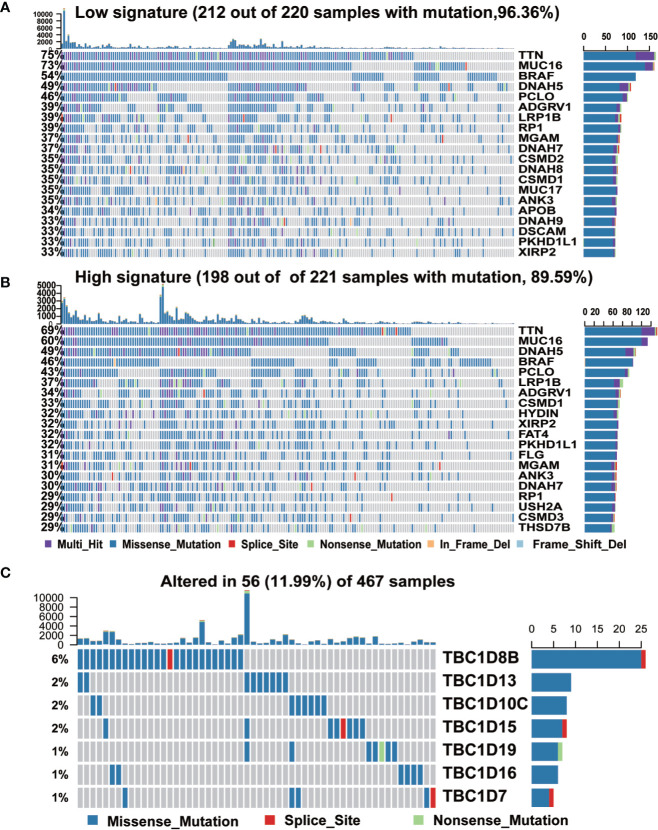
The mutation frequency of top 20 genes and seven prognostic TBC genes in melanoma. **(A, B)**. Mutated oncogenes in melanoma were distributed into low and high risk-score subgroups. The mutation frequency of top 20 genes. **(C)**. Frequency of the seven prognostic TBC genes in total samples from the TCGA dataset.

We further analyzed the occurrence of somatic mutations in the TBC genes and their influences on melanoma prognosis. Somatic mutations were observed in 212 (96.36%) and 198 (89.59%) of 221 samples in subgroups with low- and high-risk signature, respectively. Both groups shared mutations in 12 genes (TTN, MUC16, BRAF, DNAH5, ADGRV1, LPR1B, PCLO, RP1, MGAM, DNAH7, ANK3, and PKHD1L1). The frequency of the mutations in the low- and high-signature groups were 75 vs 69% for TTN, 73 vs 60% for MUC16, 54 vs 46% for BRAF, 46 vs 43% for PCLO, 39 vs 31% for ADGRV1, 39 vs 37% for LRP1B, 39 vs 29% for RP1, 37 vs 31% for MGAM, 37 vs 30% for DNAH7, 35 vs 30% for ANK3, 33 vs 32% for PKHD1L1, and the frequency of DNAH5 was identical in both groups (49%). We also observed mutations of CSMD2 (35%), DNAH8 (35%), MUC17 (35%), APOB (34%), DNAH9 (33%), and DSCAM (33%) in low signature group, and the mutation of HYDIN (32%), FAT4 (32%), FLG (31%), USH2A (29%), CSMD3 (29%), and THSD7B (29%) in the high signature group (**Figures 4A, B**). What’s more, mutations in the seven prognostic-associated TBC genes were observed in 56 (11.99%) of the 467 TCGA samples, among which TBC1D8 showed the most frequent mutation with a frequency of 6%. It’s worth noting that missense mutation was the most common type of mutation for these genes (**Figure 4C**).

### Higher Seven Prognostic-Associated TBCs Risk Score Resulted in Worse Prognosis and Metastasis in Melanoma Patients

**Figure 5:**
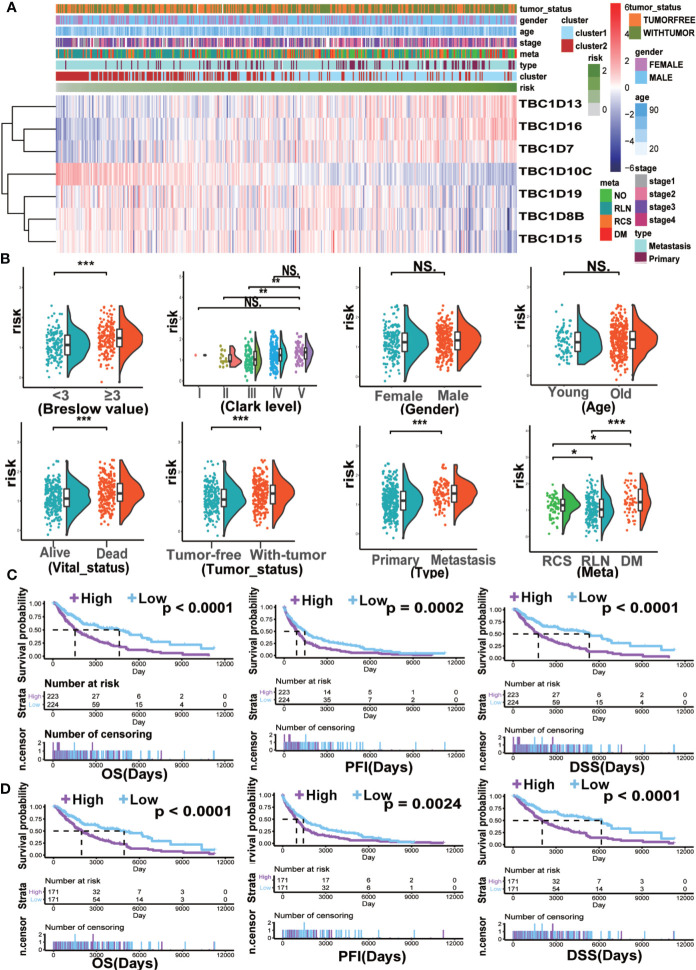
The clinical characteristics and survival analysis based on risk score. **(A)** The risk score model of the seven prognostic-associated TBCs in melanoma was constructed based on the coefficient of LASSO. The distributions of clinical characteristics and the seven prognostic-associated TBCs expression based on the risk scores are showed with heat map. **(B)** The differences in risk score within various subgroups classified by clinical features including Breslow value, Clark level, Gender, Age, Vital status, Tumor status, Tumor type and Meta in the melanoma TCGA dataset. *p < 0.05, **p < 0.01, ***p < 0.001, NS. p > 0.05. Kaplan ± Meier survival analyses demonstrated the differences in OS, PFI and DSS based on risk scores (High vs Low) in tumor tissues **(C)** and Metastasis tissues **(D)** from TCGA.

The seven prognostic-associated TBC genes screened out from the LASSO Cox regression analysis were fitted into a formula based on gene expression level to calculate the risk score with the coefficient of each gene identified by multivariate Cox regression analysis. The risk score = −0.026×exp (TBC1D8B) + 0.034×exp (TBC1D7) − 0.114×exp (TBC1D19) + 0.086×exp (TBC1D16) − 0.043 × exp (TBC1D15) + 0.279×exp (TBC1D13) − 0.151×exp (TBC1D10C). The heat map exhibited the expression patterns of seven prognostic-associated TBC genes in TCGA dataset according to the risk scores (**Figure 5A**). Comparison of the risk scores among subgroups according to clinical characteristics were shown in **Figure 5B**. A higher mean risk score with Breslow depth value ≥3 compared with Breslow depth value <3 was observed (**Figure 5B**). According to melanoma Clark levels, the risk score of Clark level V was higher than that of the Clark level II and III, respectively (**Figure 5B**). Those died patients showed a significantly higher mean risk score than the alive patients. Patients with tumor also showed higher risk scores as compared with tumor-free patients (**Figure 5B**). Metastasis patients also showed higher risk scores than the primary patients, and the risk score was the highest in patient with distant metastasis (**Figure 5B**). Melanoma patients were grouped into low- and high-risk subgroups based on the median risk score. The high-risk patients showed poor outcomes in comparison with the low-risk patients in terms of OS, PFI, and DSS (**Figure 5C**). Patients were further divided into primary and metastasis melanoma based on the tumor type. In the primary patients, no difference in OS and DSS was observed between the high and low risk groups, except for a shorter PFI in high risk patients (**Supplementary Figure 4A**). However, in patients with metastasis, the high-risk subgroup showed remarkably poorer OS, PFI, and DSS (**Figure 5D**). Similarly, patients in the high-risk subgroup showed a shorter DMS and DSS than those in the low-risk subgroup in the validation GSE dataset (GSE65904) (**Supplementary Figure 4B**). These findings indicated that the TBCs-based diagnosis model is of excellence sensitivity.

### Consensus Clustering Analysis of Melanoma Indicated Poor Prognosis in the Cluster With Higher Expression of the Prognostic-Associated TBC Genes

**Figure 6:**
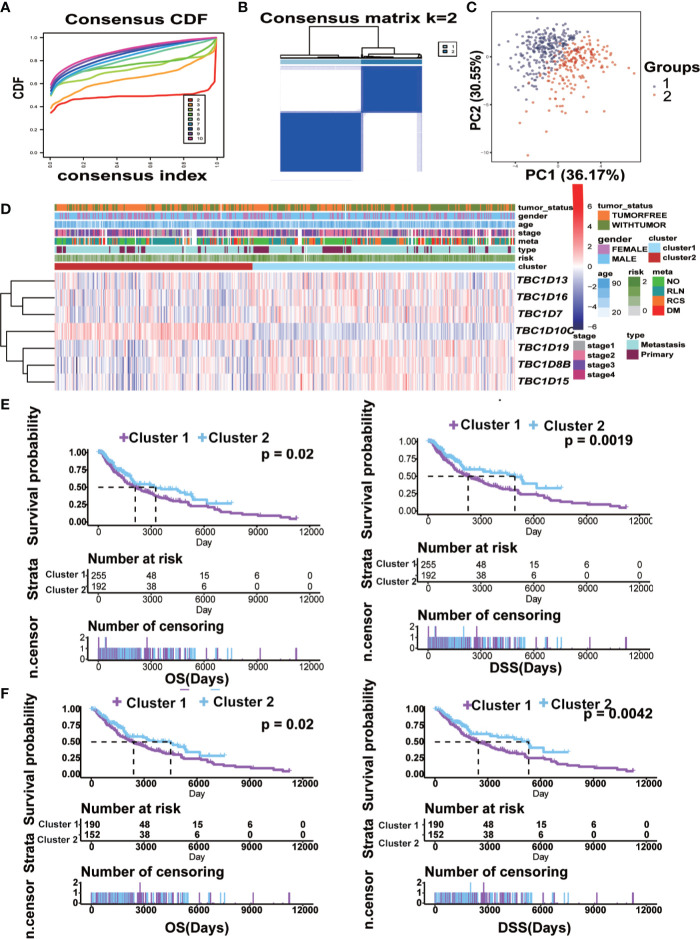
Construction of cluster model and the clinical characteristics and survival analysis based on clusters. **(A)** Clustering by the consensus clustering algorithm with k= 2 to 10. The cumulative distribution function (CDF) plot of the TBCs mRNA expression in melanoma from TCGA. k=2 was defined as the optimal number. **(B)** Consensus matrix for 2 clusters. The dark blue rectangles show the samples assigned to the 2 clusters while the light blue lines represent the unassigned samples. **(C)** The distributions of clinical features and the seven prognostic associated TBCs expression according to the clusters (cluster 1 vs cluster 2) in TCGA are showed by heat maps. **(D)** Comparisons of risk score values between subgroups separated by clinicopathological characteristics. Kaplan ± Meier survival analyses demonstrated the differences in OS and DSS based on clusters (cluster 1 vs cluster 2) in tumor tissues **(E)** and Metastasis tissues **(F)** from TCGA.

To explore the potential predictive value of TBC family members, melanoma samples were analyzed by consensus clustering analysis. The results from the cumulative distribution function (CDF) curves and consensus matrixes showed the best performance as the optimal number of clusters k was set at 2 (k=2) (**Figures 6A, B**). Principal component analysis also showed differential mRNA expression in TBC genes between the two clusters (**Figure 6C**). Clustering analysis based on the prognostic-associated TBC genes integrated with clinical traits showed that the cluster 1 was associated with tumor status (**Figure 6C**). Difference in mRNA expression of the prognostic-associated TBC genes between the two groups were also observed, specifically, a higher expression of TBC1D16, TBC1D7, TBC1D19, TBC1D8B, TBC1D15, and a lower expression of TBC1D10C was observed in cluster 1. Patients in cluster 1 showed obviously shorter OS and DSS for the TCGA melanoma dataset (**Figure 6E**), though no difference in PFI was observed (**Supplementary Figure 4C**). When stratified by primary and metastasis characteristics, no difference in OS and DSS between the clusters except for a shorter PFI in cluster 1 was observed for the primary patients (**Supplementary Figure 4E**). For the metastasis patients, cluster 1 showed obviously shorter OS and DSS (**Figure 6F**) but comparable PFI than cluster 2 (**Supplementary Figure 4D**).

### Pathway Analysis and Correlation Analysis of the Seven Prognostic-Associated TBC Genes

**Figure 7:**
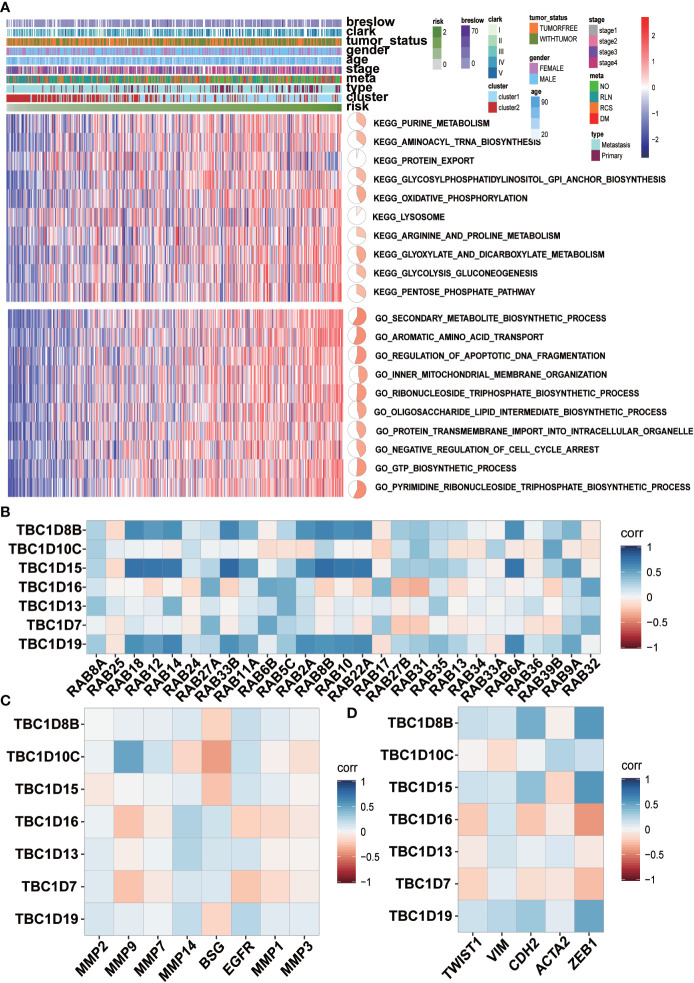
GSVA analyses and correlation analysis. **(A)** GSVA analyses for the seven prognostic-associated TBCs. The distribution of risk scores, clinical characteristics and gene set enrichment of different pathways was displayed by heatmap. **(B)** Correlation analysis between the seven prognostic TBCs and several important RAB family **(C)** Correlation analysis between the seven prognostic TBCs and and invasion related genes (MMP2, MMP9, MMP7, MMP14, BSG, EGFR, MMP1, and MMP3). **(D)** Correlation analysis between the seven prognostic TBCs and EMT associated genes (TWIST1, VIM, CDH2, ACTA2, and ZEB1).

GSVA analysis was conducted to investigate the possible biological functions of the TBC genes in melanoma. The top 10 signaling pathways with significant differences were selected from GO and KEGG analysis. Majority of these associated pathways are involved in the metabolism of signaling molecules such as purine metabolism, aminoacyl tRNA biosynthesis, glycosylphosphatidylinositol GPI anchor biosynthesis, oxidative phosphorylation, glycolysis and dicarboxylate. Some pathways were involved in transportation such as protein export, lysosome, aromatic amino acid transport, inner-mitochondrial membrane organization, protein transmembrane import into intracellular organelle. Also, pathways related to the regulation of apoptotic DNA fragmentation and negative regulation of cell cycle arrest were also observed (**Figure 7A**). Potential biological function analysis of the seven prognostic-associated *TBC* genes showed that *TBC1D16*, *TBC1D13* and *TBC1D7* were strongly correlated with pathways described above (**Supplementary Figures 5A, B**).

The main function of Rab proteins is to recruit effector molecules to promote the activation of downstream traffic events. Therefore, we analyzed the correlation of mRNA expression of the seven prognostic-associated TBC genes with 27 Rab family genes reported by literatures (**Figure 7B**). Strong correlations between the prognostic-associated TBCs and RAB genes were observed. Positive correlation between TBC1D18 and 10 Rab genes (RAB18, RAB12, RAB14, RAB33B, RAB11A, RAB2A, RAB8R, RAB10, RAB22A, and RAB6A) was observed. TBC1D15 also correlated with 10 Rab genes (RAB18, RAB12, RAB14, RAB33B, RAB11A, RAB2A, RAB8B, RAB10, RAB22A, and RAB6A). TBC1D19 correlated extensively with 10 of the Rab genes (RAB18, RAB12, RAB14, RAB33B, RAB11A, RAB2A, RAB8B, RAB10, RAB22A, and RAB6A).

Correlations between the 7 prognostic-associated TBC genes and invasion-related genes including BSG, EGFR, matrix metalloproteinases (MMP2, MMP7, MMP9, MMP14, MMP1, and MMP3) and EMT-related genes (TWIST1, VIM, CDH2, ACTA2, and ZEB1) were also analyzed. Specifically, TBC1D10C was correlated positively with MMP9, MMP7 and EGFR. TBC1D16 correlated positively with MMP14 and BSG. TBC1D13 was correlated positively with MMP2, MMP14, BSG and EGFR. TBC1D19 was correlated positively with MMP2, MMP14 and EGFR (**Figure 7C**). In addition, TBC1D8B, TBC1D10C, TBC1D15, TBC1D13, and TBC1D19 were correlated positively with TWIST1, VIM, CDH2, ACTA2, and ZEB1, respectively (**Figure 7D**).

### Associations Between the Seven Prognostic-Associated TBC Genes With Immune Characteristics

**Figure 8:**
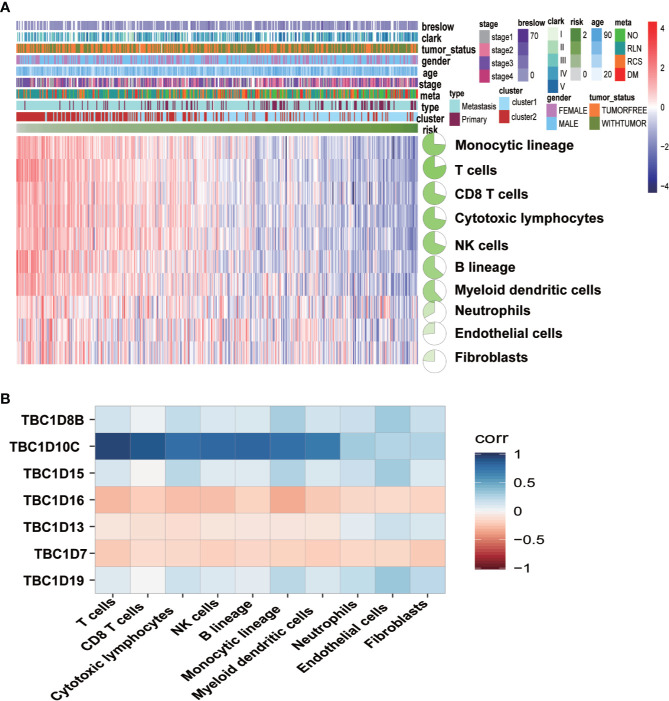
Associations between the seven prognostic-associated TBC genes with immune characteristics. **(A)** The relationship of the distributions of clinical features and immune cells in TCGA is showed by heat maps. **(B)** Correlation analysis between the seven prognostic TBCs and immune cells.

To determine whether there was correlation between the seven prognosis-associated TBC genes and immune cells, a further analysis with immune cells including monocytic lineage, T cells, CD8 T cells, cytotoxic lymphocytes, NK cells, B lineage, myeloid dendritic cells, neutrophils, endothelial cells plus fibroblasts were analyzed. Differential expression profiles of immune cells in melanoma from the TCGA dataset was indicated by the heatmap (**Figure 8A**). Correlation between the seven prognostic-associated TBC genes with the immune cells was shown in **Figure 8B**. The results showed TBC1D8B, TBC1D10C, TBC1D15, and TBC1D19 were correlated with immune signatures. Interestingly, TBC1D10C was found to be correlated with T cells, CD8 T cells, cytotoxic lymphocytes, NK cells, B lineage, monocytic lineage and myeloid dendritic cells. Only weak correlations between TBC1D16, TBC1D13, TBC1D7, and immune cells were observed.

### Prognostic Nomogram Model Constructed Based on Age, Stage, Risk Score, and Primary Focus Predicted OS in Melanoma

**Figure 9:**
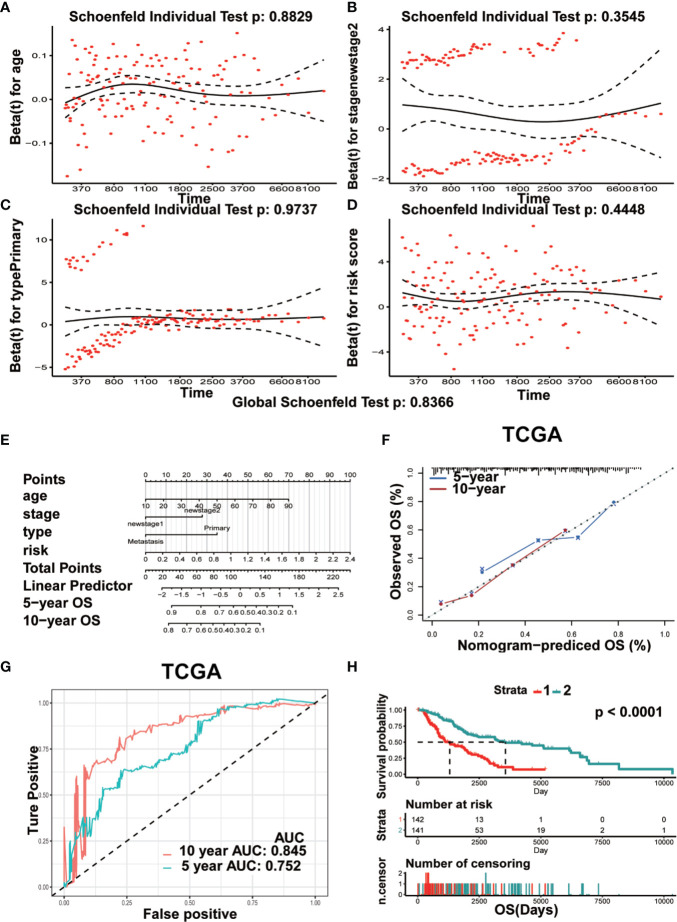
Prognostic nomogram model was constructed for predicting OS. **(A–D)** The plots of Schoenfeld residual were showed for age **(A)** stage II **(B)** Primary **(C)** risk **(D)** in the prognostic nomogram. **(E)** The prognostic nomogram for melanoma patients was established dependent on four crucial factors. **(F)** The calibration curve of OS predicted by nomogram. The predicted possibility of OS is responded to x-axis and the observed OS is responded to the y-axis. **(G)** The curve of ROC based on the prognostic nomogram at 5-year and 10-year OS. **(H)** Comparison of OS between two groups (Strata 1 vs Strata 2).

Univariate and multivariate Cox analyses were used to assess the independent prognostic index associated with the clinical outcomes of patients. Results of multivariate Cox analysis showed that age (HR=1.02, P=0.0015), stage 2 (HR=1.76, P=0.0019), risk score (HR=2.43, P=7.42E-6), and primary focus (HR=1.87, P=0.0413) were independent prognostic indicators for OS. The Clark level II and Breslow value showed significance in the univariate Cox analysis but lost in the multivariate Cox analysis. No remarkable gender difference was observed (**Supplementary Table 2**). The results of **Figures 9A–D** showed the Schoenfeld tests value (Global Schoenfeld test = 0.8366, Age, *p* = 0.8829; Stage2, *p* = 0.3545; Primary, *p* = 0.9737; Risk score, *p* = 0.4448), indicating that each variable met the requirements for proportional hazards test (PH) (PH>0.05). Four significant prognostic variables identified in the Cox regression model were introduced to construct the nomogram model. As shown in **Figure 9E**, each factor was given a score on the axis, the points of all variables were joined together and the outcome was depended on the position on the survival axis based on the total score. Calibration curves confirmed the degree of precision of the nomogram, and the OS predicted by the nomogram was in accordance with 5-year OS and 10-year OS (**Figure 9F**). The AUC values under the ROC curve were 0.752 and 0.845, respectively, in predicting the 5-year and 10-year OS for melanoma patients (**Figure 9G**). Survival analysis showed a significant difference in term of OS between the two groups (p< 0.0001) (**Figure 9H**).

### Interference of TBC1D7 Expression Inhibits Melanoma Cell Proliferation, Migration, and Invasion

**Figure 10:**
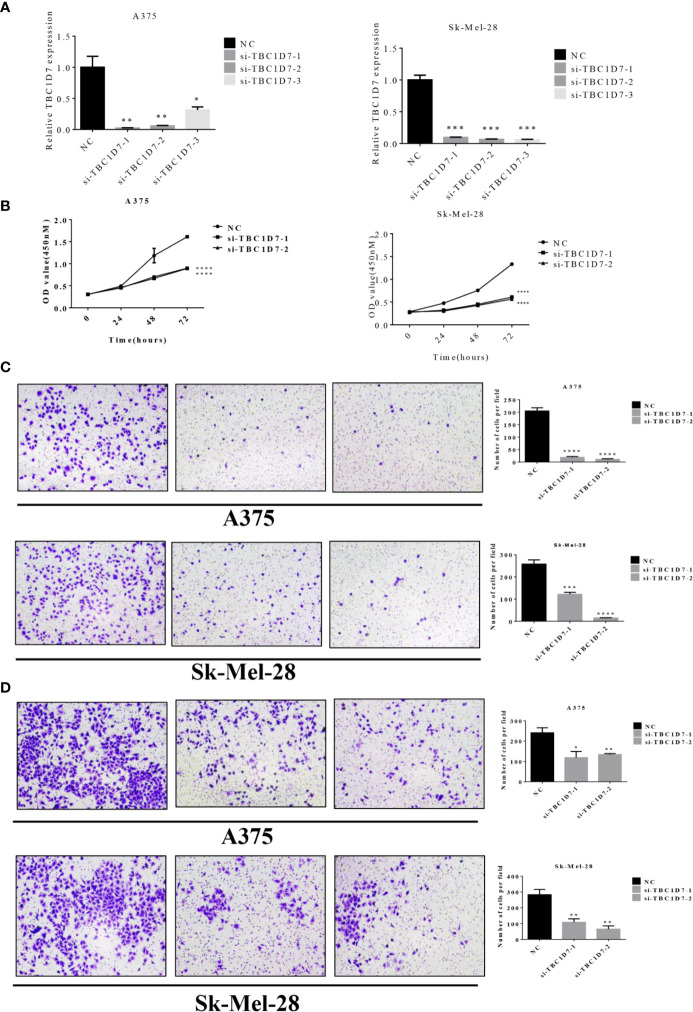
Interference of TBC1D7 expression inhibits melanoma cell migration and invasion. **(A)** The relative mRNA levels of TBC1D7 at 36 h after transfection with si-TBC1D7-1, si-TBC1D7-2, si-TBC1D7-3 and NC as control in melanoma cells A375 and Sk-Mel-28 were measured by RT-PCR. GAPDH was used as a control. *p <, **p < 0.01, ***p < 0.001. **(B)** Melanoma cells A375 and Sk-Mel-28 interfered with si-TBC1D7, 2.0 x 103 cells planted into 96-well cultured for 24, 48, 72 h, CCK-8 kits were used to detect the proliferation at OD450nM following the manufacturer’s instruction. Each group have 5 replicates(n=5) with 3 independent experiments. ****p < 0.0001. **(C)** A number of 1 x 10^4^ cells were seeded into the upper chambers with 550ul medium containing 30% FBS put into the lower chambers. Chambers after cultured for 24 h were fixed with 4% paraformaldehyde, stained with crystal violet and imaged by microscope. Representative images of migration of A375 and Sk-Mel-28, the numbers of magration cells per field were calculated, and the results are presented as the mean ± SD (n = 3). ***p < 0.001, ****p < 0.0001. **(D)** 5 x 10^4^ cells were seeded in the chambers embed with matrigel cultured for 30 h and performed as material and methods. Representative images of invasion of A375 and Sk-Mel-28, the numbers of invasion cells per field were also calculated. *p < 0.05, **p < 0.01.

In order to further investigate the function role of TBC proteins in melanoma, TBC1D7, one of TBC proteins was chosen for subsequent functional validation. As shown in **Figure 10A**, TBC1D7 mRNA expression in both melanoma cell lines A375 and Sk-Mel-28 were successfully interfered with siRNA which was confirmed by RT-PCR, and two sequences of them were selected to explore the effect of TBC1D7 on melanoma cells. TBC1D7 deficiency induced by siRNA suppressed the proliferation of melanoma cells A375 and Sk-Mel-28 obviously (**Figure 10B**). To further determine whether TBC1D7 could inhibit the metastasis of melanoma, Boyden chambers were used to assess melanoma cell migration and invasion. The results showed that depletion of TBC1D7 in both melanoma cell lines A375 and Sk-Mel-28 impeded the ability of migration (**Figure 10C**). Similarly, suppression of TBC1D7 with siRNA also inhibited the invasion of melanoma cells remarkably (**Figure 10D**). Collectively, these results suggested that TBC1D7 inhibits the migration and invasion ability of melanoma cells.

## Discussion

Melanoma is a highly mutated and metastatic cancer originated from the malignant transformation of melanocytes. Men are at a 40% increased risk in their lifetime to be diagnosed with melanoma (21). As the incidence of melanoma progressively increasing, illuminating the complicated pathogenesis and risk factors are crucial. TBC family members are classical GAPs negatively regulating the hydrolysis of GTP, the latter is engaged in the cycles of RABs (22). TBCs are involved in the pathogenesis of various diseases such as dermatitis, obesity, bacterial infection and tumorigenesis (8). Some TBCs may act as oncogenes that affect disease survival and metastasis. For example, TBC1D3 is observed to be overexpressed in breast cancer by promoting oxidized low-density lipoprotein receptor 1 expression required for cell migration involving in TNFα/NF-κB signaling (23). Daigo Y et al. discovered the activation of TBC1D7 in lung cancer and suppression of TBC1D7 inhibited the growth of lung cancer cells (12). However, few studies have focused on functions of TBC family members in melanoma.

In this study, we conducted a comprehensive analysis of the characteristics of 33 *TBC* genes in melanoma. We firstly analyzed the relationship between mRNA expression of *TBC* genes and clinical features such as age, gender, tumor type and stage in melanoma. We observed a strong correlation between mRNA expressions of some of TBC genes such as *TBC1D10C, TBC1D4, TBC1D23* and clinical-pathological features (**Figure 2**). Seven prognosis associated TBCs genes including *TBC1D13*, *TBC1D16*, *TBC1D7*, *TBC1D10C*, *TBC1D19*, *TBC1D8B*, and *TBC1D15* were identified in melanoma. Few studies have focused on the roles of these *TBC* genes in melanoma. TBC1D13 is a specific GAP of RAB35 that plays a crucial effect on the trafficking of GLUT4 in adipocytes (24). Interestingly, the role of TBC1D13 in melanoma remains unknown. Evidence has shown that TBC1D16 is a driver of melanoma (13). Hypomethylation of TBC1D16 leads to the activation of TBC1D16 transcription in melanoma, and the short isoform of TBC1D16 (TBC1D16-47KDa) promotes melanoma growth and metastasis through interacting with RAB5C and regulating EGFR signaling (25). Knockdown of TBC1D7 resulted in the activation of mTORC1 signaling, and enhanced cell growth (26). Interests in TBC1D10C are mainly focused on immune response (27, 28). Two pieces of literature reported TBC1D19, one showed that TBC1D19 acted upon cell polarity and decreased TBC1D19 expression contributed to the disruption of odontoblast polarity and apoptosis (29). Interference of TBC1D8B increased basal autophagy and exocytosis through inhibiting the expression of RAB11 which plays a crucial role in the pathogenesis of nephrotic syndrome (30). TBC1D15 controlled glucose uptake through RAB7 in late endosomal pathway impacting GLUT4 translocation (31). Bibliography retrieval on above genes showed that the function of these *TBC* genes is unclear, the role of these TBCs in the melanoma is largely unknown. Somatic alteration analysis showed genomic alterations in both the high- and low-risk groups, and the mutant frequency of *TBC1D8* is the highest among the seven TBC genes (**Figure 4**). Hemizygous missense mutations in *TBC1D8B* have also been reported in families with nephrotic syndrome (30). However, little is known about the functional significance of genomic alteration of these TBCs.

We used LASSO Cox regression analysis to calculate the risk score according to the seven most meaningful TBC genes in this study. We observed a higher risk score related to clinical-pathological characteristics of melanoma such as Breslow value (>3), Clark level IV, V, vital status (dead), tumor status (with tumor), tumor type (metastasis). In addition, a higher risk score was also associated with shorter OS and DSS (**Figure 5**). Consensus clustering analysis grouped the patients into two clusters, the principal component analysis further demonstrated differential expression of the TBC genes between the two clusters. Cluser1 showed shorter OS and DSS (**Figure 6**). The potential function of TBC genes in melanoma may include vesicular transport such as protein export, amino acid transport, and protein transmembrane import (**Figure 7**). Take TBC1D15 as an example, Ishihara N et al. reported that Fis1 acts as a mitochondrial recruitment factor for TBC1D15 which is engaged in the regulation of mitochondrial morphology (32). Senga T et al. discovered that knockdown of TBC1D15 resulted in the activation of RhoA and membrane blebbing, which was blocked by inhibiting RhoA signaling (33). TBC1D15 acts as a GAP of Rab7 in regulation of the lysosomal morphology (34). Aberrant intracellular transport is involved in type 2 diabetes, immune deficiencies and cancer (35),. Besides, correlation analysis indicated a positive correlation with signal molecule synthesis, apoptosis and cell cycle arrest (36, 37). TBC/RABGAPs regulate the malignant behavior involved in the regulation of RABs. Nearly a half of RABs have been identified as substrates for TBC/RABGAP family members. Results of correlation analysis for the seven prognostic associated TBCs and RABs in our study indicated that the same TBC gene might be regulated by various RABs, and the same RAB gene might be regulated by different TBCs. TBC1D8B is observed to regulate autophagy and exocytosis interacting with RAB11 (30). As could be seen from **Figure 7**, TBC1D8B might be regulated by RAB18, RAB12, RAB14, RAB33B, RAB2A, RAB8B, RAB10, RAB22A, RAB17 except for RAB11A. Meanwhile, RAB18 might be regulated by TBC1D8B, TBC1D15, TBC1D19, which gave us hints that the complex mechanism of TBCs regulated by RABs. We also investigated the correlation of the seven prognostic-associated TBCs between MMP-related genes (MMP2, MMP9, MMP7, MMP14, BSG, EGFR, MMP1, MMP3) and EMT-associated genes (TWIST1, VIM, CDH2, ACTA2, ZEB1), which also indicated a central role of TBCs in melanoma. As reported in the literature, TBC1D16 as a driver of melanoma is a Rab4A GAP that is engaged in the trafficking of transferrin receptor and EGFR (38). TBC1D8B can interact with RAB11B that is involved in vesicular recycling (30, 39).

Immune microenvironment is also key important for tumors (40–42). Melanoma patients are supposed to develop immune responses against specific tumor antigens (43). Therefore, We also investigate the role of immune cells in melanoma, and correlation analysis of the seven prognostic-associated TBC genes with immune cells showed that differential variations of the seven genes regarding immune cells, especially for TBC1D10C (**Figure 8**). As reported, TBC1D10C is identified to be related with immune response in head and neck squamous cell carcinoma (27), and artificial neural networks analysis also confirmed the involvement of TBC1D10C in immune response through TCGA (28), suggesting the possible mechanisms of these TBCs in regulation of the immune microenvironment.

We also constructed a prognostic nomogram for predicting OS of melanoma and found the independent prognostic indicators such as age, risk score based on the expression levels of the seven TBC genes, stage 2 and primary focus (**Figure 9**). What’s more, TBC1D7, one of the seven TBC proteins was chosen for further experimental validation, TBC1D7 is the third subunit of TSC1/TSC2 associated with autophagy (26). It has been reported that TBC1D7 is related with various diseases such as intellectual disability, megalencephaly (44), diabetes (45) and tumor (12). Highly expression of TBC1D7 was related with poor outcome in non-small cell lung cancer (NSCLC), and suppression of TBC1D7 inhibits the growth of lung cancer cells, which might be a promising target in cancer therapy (12). By using mass spectrometry-based quantitative proteomic method, a recent study by Qi et al. observed that TBC1D7 is a potential driver for melanoma cell invasion by transwell assay, and higher TBC1D7 expression is associated with poor outcome as analyzed by the TCGA and GEO (GSE65904) data (46). In our study, we also observed that interference of TBC1D7 expression inhibited the proliferation, migration and invasion of melanoma cells A375 and Sk-Mel-28 *in vitro* (**Figure 10**), suggesting the potential role of TBC1D7 in melanoma. Different from the study by Qi et al., we investigated the association of mRNA expression of TBCs and the clinical traits in melanoma, and we observed that in spite of TBC1D7, six other prognostic-associated TBC genes TBC1D13, TBC1D16, TBC1D7, TBC1D8B, TBC1D15, TBC1D19, and TBC1D10C were also identified by LASSO Cox regression analysis In addition, mutations profile of these genes was also analyzed. A risk score model, cluster model and prognostic nomogram model which were well established to predict the OS. What’s more, the pathway analysis and correlation analysis of the seven prognostic-associated TBC genes with RAB proteins, invasion-associated genes (i.e *BSG*, *EGFR* and *MMP*) and EMT-associated genes (i.e *TWIST1*, *VIM*, *CDH2*, *ACTA2*, and *ZEB1*) were also explored. Immune microenvironment is another key factor promoting the malignant phenotype of melanoma, and we also conducted an analysis to understand the relationship between the seven prognostic-associated TBC genes and immune cells hoping for finding clues under the view of immune microenvironment for melanoma. However, more experiments needed to be performed to investigate the detailed mechanism of TBC1D7 in melanoma cells, and the biological function of the seven prognostic-associated TBCs in melanoma is still largely unknown, further study should be clarified the effect of TBCs on melanoma.

## Conclusion

This study identified seven prognostic-associated TBCs based on comprehensive bioinformatics analysis of TBCs expression profiles and clinical features. The prognostic model of the seven prognostic-associated TBCs showed a good performance in the prediction of survival. Correlation between the prognostic-associated TBCs and RAB family members, invasion-related genes and immune cells are also observed. The nomogram integrating the seven prognostic-associated TBCs and clinical features could be more accurate in predicting the survival of melanoma patients. What’s more, Interference of TBC1D7 was confirmed to inhibit the migration and invasion in melanoma cells *in vitro*, indicating the potential therapeutic role of TBC proteins in melanoma.

## Data Availability Statement

The original contributions presented in the study are included in the article/**Supplementary Material**. Further inquiries can be directed to the corresponding authors.

## Author Contributions

XC, X-PC, and CP conceived and designed the idea for the manuscript and financial support. LT was responsible for writing this paper. S-SZ, ZZ, and HL contributed the collection and interpretation of data. QC provided analysis tools and analyzed the data. All authors contributed to the article and approved the submitted version.

## Funding

This work was supported by the Major Projects of International Cooperation and Exchanges NSFC (grant number 81620108024) and the National Natural Science Foundation (grant number 81572679, 81430075, 81673518), National Science and Technology Major Project (grant number 2013zx09509107), and the Independent Exploration and Innovation Project of Central South University in China (grant number 2019zzts344).

## Conflict of Interest

The authors declare that the research was conducted in the absence of any commercial or financial relationships that could be construed as a potential conflict of interest.

## Supplementary Material

Supplementary Figure 1 Relationship between TBCs mRNA expression with clinical features in melanoma. The heat maps based on clustering analysis according to the subgroups exhibited the differential expression patterns of TBCs (Tumor status: tumor-free vs with-tumor, Metastasis type: RCS vs RLN vs DM and Age: young vs old). *p < 0.05, **p < 0.01, ***p < 0.001, **** < 0.0001.

Supplementary Figure 2 The differential expression of the seven prognostic-associated TBC genes. The differential expression of the seven prognostic-associated TBC genes between normal tissues and tumor tissues in the TCGA dataset. ***p <0.001, NS. p > 0.05.

Supplementary Figure 3 Protein expression of TBCs detecting by immunohistochemical assay based on online websites. Immunohistochemical staining showed the images of the protein expression of TBC1D10C **(A)**, TBC1D19 **(B)**, TBC1D16 **(C)**, and TBC1D13 **(D)** in normal skin tissues and melanoma tissues from the Human Protein Atlas website (www.proteinatlas.org).

Supplementary Figure 4 Survival analyses based on risk score and clusters. **(A)** Kaplan ± Meier survival analyses demonstrated the differences in OS, PFI and DSS based on risk scores (High vs Low) in primary tissues from TCGA. **(B)** Kaplan ± Meier survival analyses demonstrated the differences in DMS and DSS based on risk scores (High vs Low) in tumor tissues from the GEO dataset (65904). **(C)** Kaplan ± Meier survival analyses demonstrated the differences in PFI based on clusters (cluster 1 vs cluster 2) in tumor tissues. **(D)** Kaplan ± Meier survival analyses demonstrated the differences in PFI based on clusters (cluster 1 vs cluster 2) in metastasis tissues. **(E)** Kaplan ± Meier survival analyses demonstrated the differences in OS, PFI and DSS based on clusters (cluster 1 vs cluster 2) in primary tissues.

Supplementary Figure 5 GO and KEGG analysis. **(A, B)** Correlation analysis between the seven prognostic TBCs and the top 10 significant pathways from GO and KEGG analysis.
