# Supplementary figures and images for: Tre2-Bub2-Cdc16 Family Proteins Based Nomogram Serve as a Promising Prognosis Predicting Model for Melanoma
Source: Front Oncol. 2020 Oct 28;10:579625. doi: 10.3389/fonc.2020.579625 (PMC7656061; doi:10.3389/fonc.2020.579625)

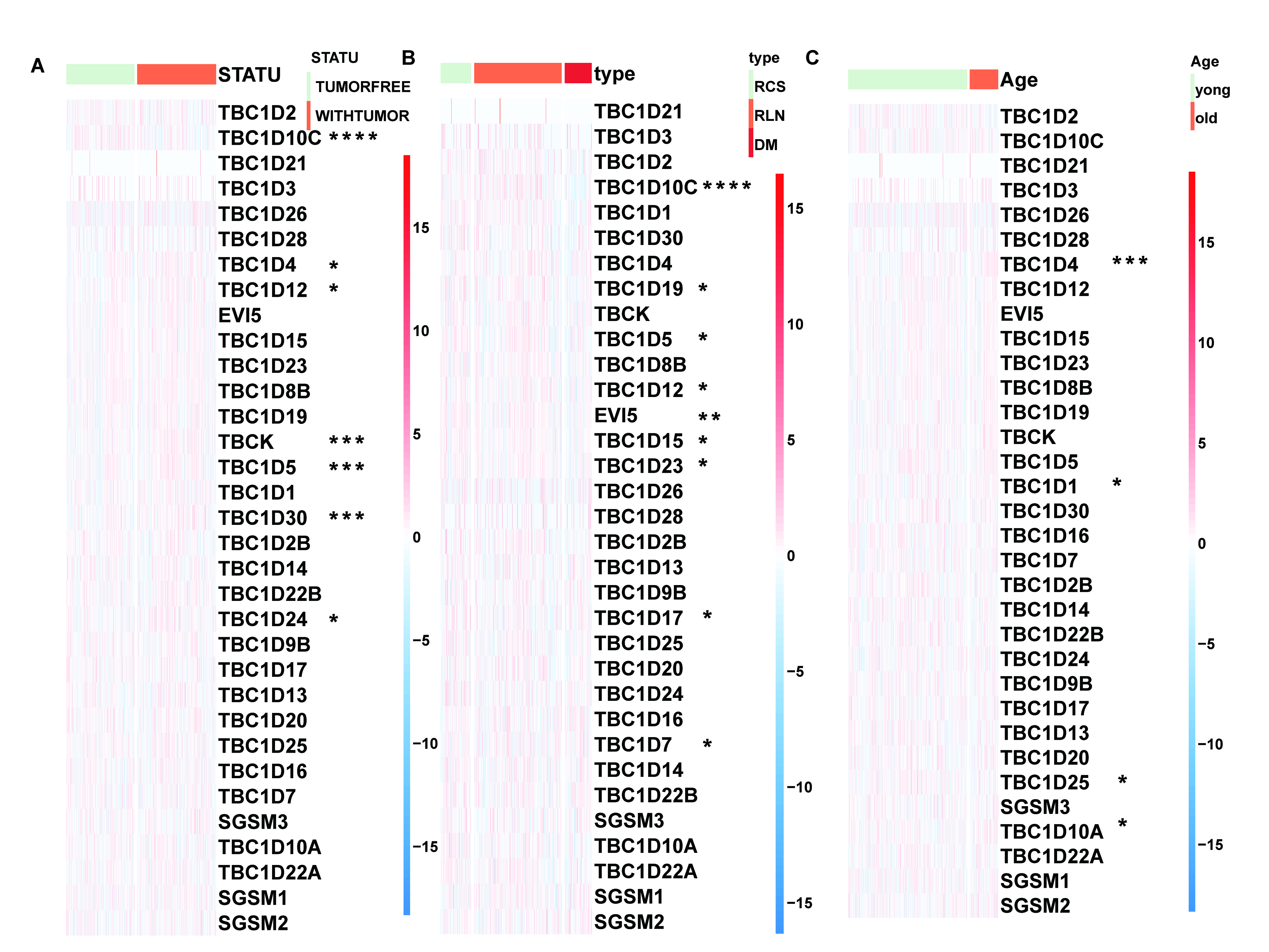

Supplement: Supplementary Figure 1 — Relationship between TBCs mRNA expression with clinical features in melanoma. The heat maps based on clustering analysis according to the subgroups exhibited the differential expression patterns of TBCs (Tumor status: tumor-free vs with-tumor, Metastasis type: RCS vs RLN vs DM and Age: young vs old). *p < 0.05, **p < 0.01, ***p < 0.001, **** < 0.0001. [file Image_1.tif]

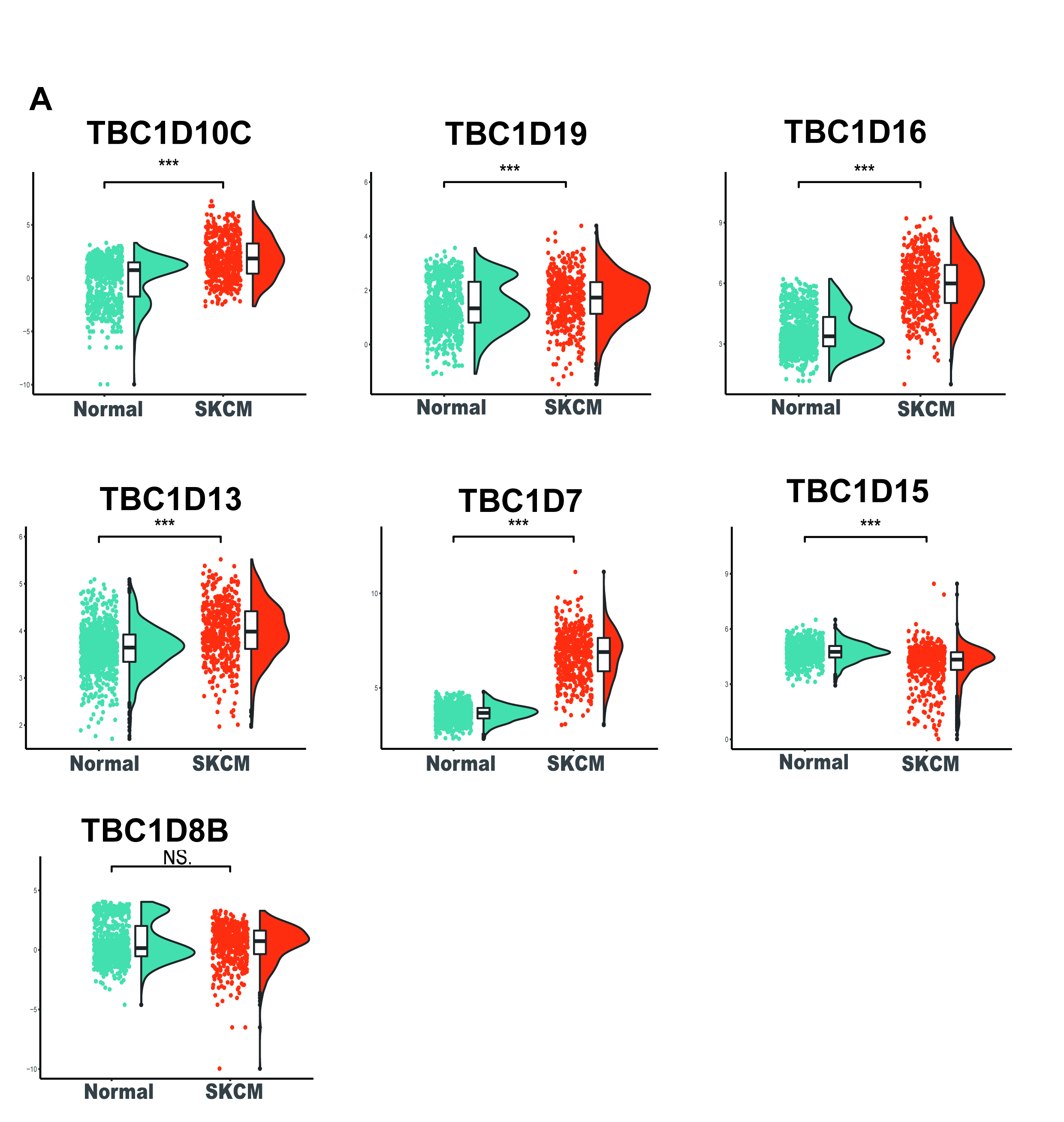

Supplement: Supplementary Figure 2 — The differential expression of the seven prognostic-associated TBC genes. The differential expression of the seven prognostic-associated TBC genes between normal tissues and tumor tissues in the TCGA dataset. ***p <0.001, NS. p > 0.05. [file Image_2.tif]

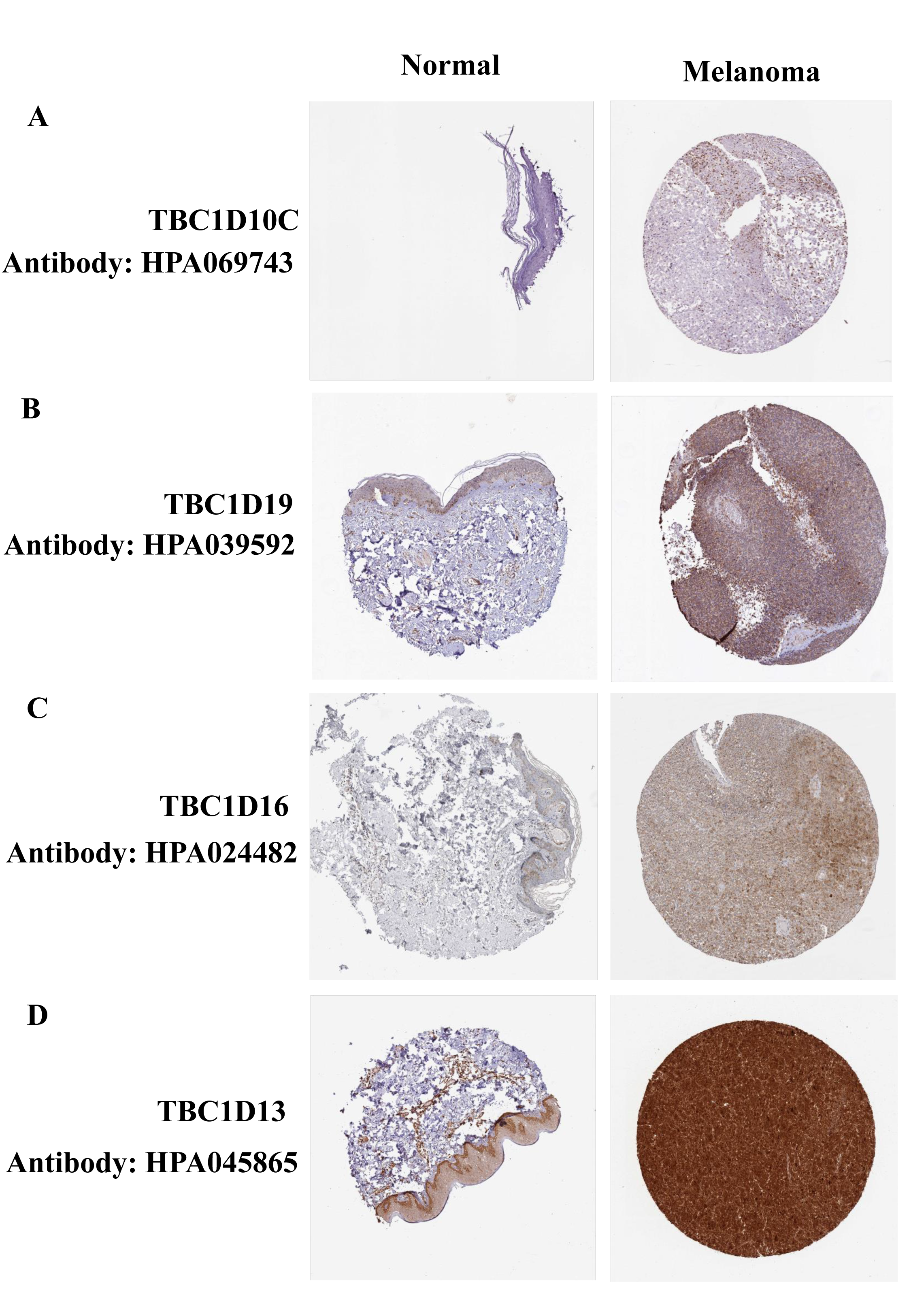

Supplement: Supplementary Figure 3 — Protein expression of TBCs detecting by immunohistochemical assay based on online websites. Immunohistochemical staining showed the images of the protein expression of TBC1D10C (A), TBC1D19 (B), TBC1D16 (C), and TBC1D13 (D) in normal skin tissues and melanoma tissues from the Human Protein Atlas website (www.proteinatlas.org). [file Image_3.tif]

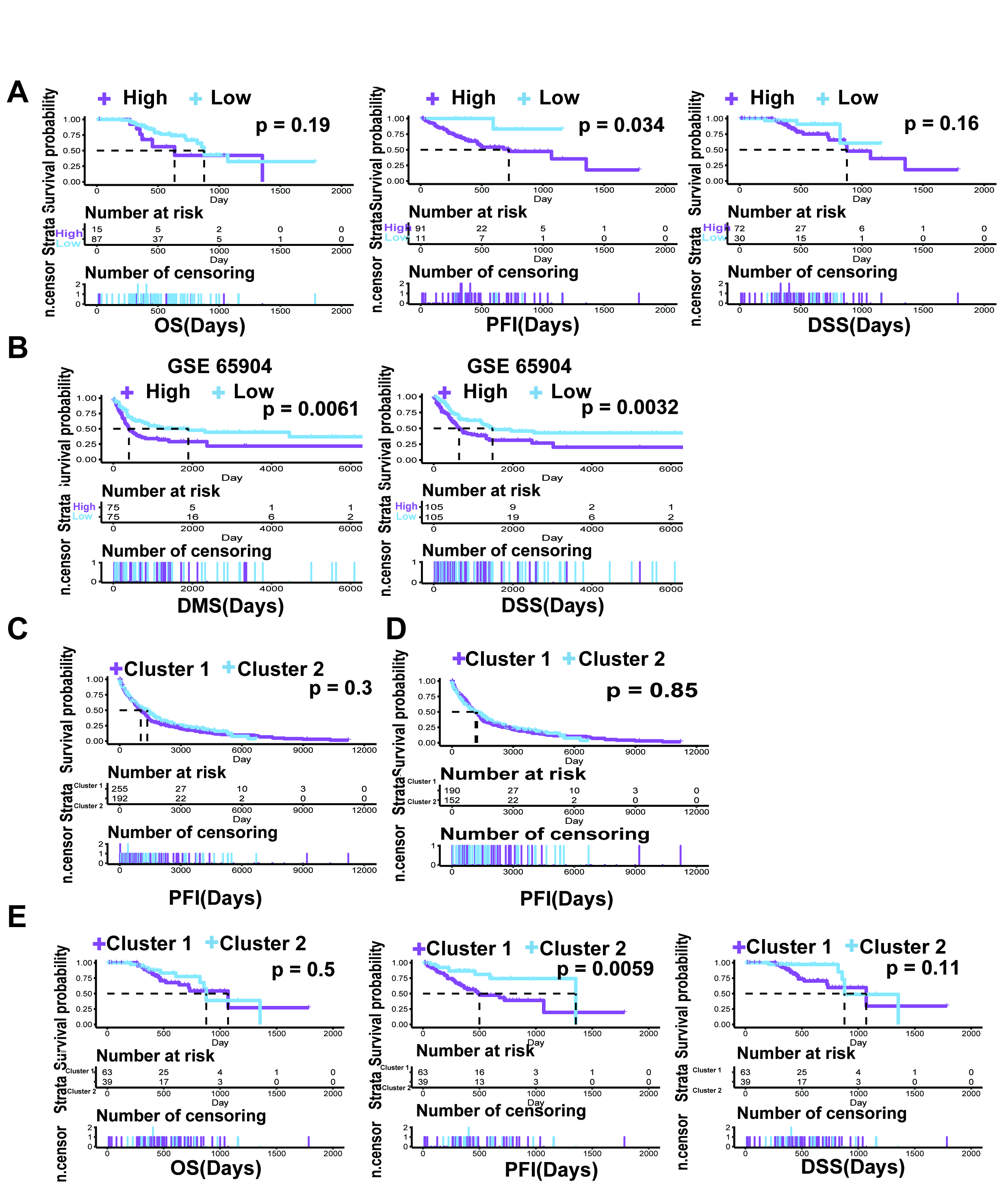

Supplement: Supplementary Figure 4 — Survival analyses based on risk score and clusters. (A) Kaplan ± Meier survival analyses demonstrated the differences in OS, PFI and DSS based on risk scores (High vs Low) in primary tissues from TCGA. (B) Kaplan ± Meier survival analyses demonstrated the differences in DMS and DSS based on risk scores (High vs Low) in tumor tissues from the GEO dataset (65904). (C) Kaplan ± Meier survival analyses demonstrated the differences in PFI based on clusters (cluster 1 vs cluster 2) in tumor tissues. (D) Kaplan ± Meier survival analyses demonstrated the differences in PFI based on clusters (cluster 1 vs cluster 2) in metastasis tissues. (E) Kaplan ± Meier survival analyses demonstrated the differences in OS, PFI and DSS based on clusters (cluster 1 vs cluster 2) in primary tissues. [file Image_4.tif]

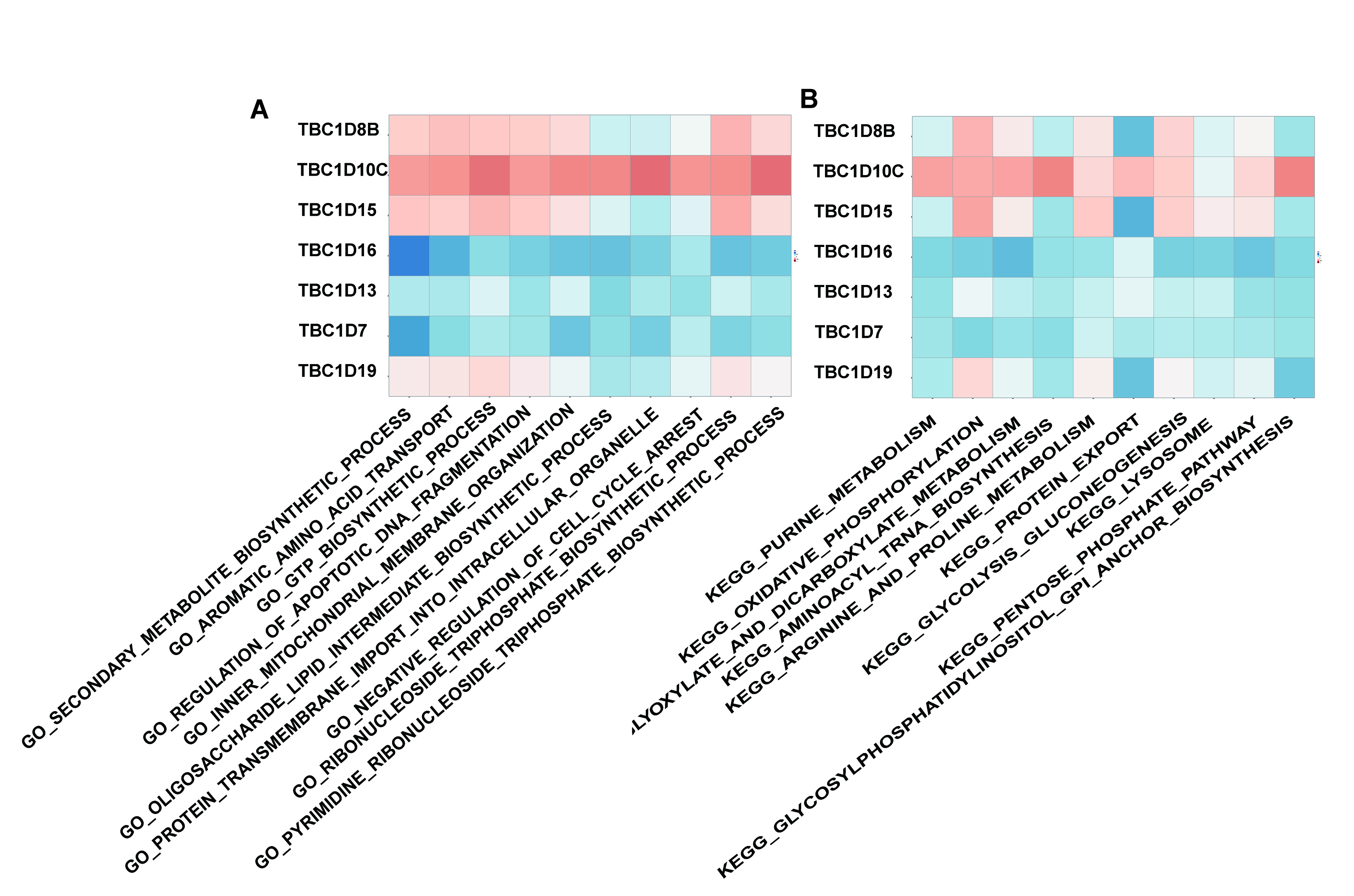

Supplement: Supplementary Figure 5 — GO and KEGG analysis. (A, B) Correlation analysis between the seven prognostic TBCs and the top 10 significant pathways from GO and KEGG analysis. [file Image_5.tif]
