# Supplementary material for: Tre2-Bub2-Cdc16 Family Proteins Based Nomogram Serve as a Promising Prognosis Predicting Model for Melanoma
Source: Front Oncol. 2020 Oct 28;10:579625. doi: 10.3389/fonc.2020.579625 (PMC7656061; doi:10.3389/fonc.2020.579625)
Supplement: Supplementary file 7 [file Table_2.docx]

**Supplementary Table 2. Univariate and multivariate Cox regression analysis of factors affecting overall survival of patients.**

| Variables | | TCGA | | | |
| --- | --- | --- | --- | --- | --- |
|  |  | Univariate analysis | | Multivariate analysis | |
|  |  | P value | HR (95%CI) | P value | HR (95%CI) |
| Age | OS | 0.0002 | 1.02(1.01-1.03) | 0.0015 | 1.02(1.01-1.03) |
|  | PFI | 0.0004 | 1.02 (1.01-1.03) | 0.0003 | 1.02 (1.01-1.03) |
|  | DSS | 0.0018 | 1.02 (1.01-1.03) | 0.0087 | 1.02 (1.01-1.03) |
| Gender(male) | OS | 0.7490 | 1.06(0.75-1.50) | 0.5772 | 1.11 (0.77-1.56) |
|  | PFI | 0.6547 | 1.07 (0.80-1.43) | 0.4427 | 1.12 (0.83-1.52) |
|  | DSS | 0.7281 | 1.07 (0.74-1.53) | 0.6398 | 1.09 (0.75-1.59) |
| TNM stage(II) | OS | 0.0010 | 1.78 (1.26-2.51) | 0.0019 | 1.76(1.23-2.52) |
|  | PFI | 1.42E-5 | 1.90(1.42-2.53) | 3.05E-5 | 1.91 (2.41-2.59) |
|  | DSS | 0.0009 | 1.83 (1.28-2.62) | 0.0018 | 1.81 (1.25-2.63) |
| Tumor type  (primary) | OS | 0.0001 | 2.87 (1.67-4.96) | 0.0413 | 1.87 (1.02-3.43) |
|  | PFI | 0.6993 | 1.10 (0.68-1.77) | 0.1253 | 0.64(0.36-1.13) |
|  | DSS | 0.0112 | 2.20 (2.20-4.01) | 0.2670 | 1.46(0.75-2.86) |
| Risk score | OS | 4.63E-8 | 2.79(1.93-4.02) | 7.42E-6 | 2.43 (1.65-3.59) |
|  | PFI | 0.0006 | 1.75(1.27-2.41) | 0.0079 | 1.57 (1.13-2.20) |
|  | DSS | 2.79E-8 | 2.95(2.01-4.32) | 1..63E-6 | 2.67 (1.79-4.00) |
| Clark level(II) | OS | 0.0011 | 1.88 (1.27-2.76) | 0.5685 | 1.13 (0.74-1.72) |
|  | PFI | 0.0096 | 1.50 (1.10-2.04) | 0.8804 | 1.03 (0.73-1.44) |
|  | DSS | 0.0026 | 1.83 (1.23-2.71) | 0.6420 | 1.11 (0.71-1.71) |
| Breslow value | OS | 0.0007 | 1.02(1.01-1.04) | 0.1562 | 1.01 (1.00-1.03) |
|  | PFI | 0.0012 | 1.02 (1.01-1.04) | 0.0027 | 1.03 (1.01-1.04) |
|  | DSS | 0.0162 | 1.02 (1.00-1.04) | 0.3195 | 1.01(0.99-1.03) |
